# Predictive modeling of response to repetitive transcranial magnetic stimulation in treatment-resistant depression

**DOI:** 10.21203/rs.3.rs-4396926/v1

**Published:** 2024-05-23

**Authors:** Lindsay Benster, Cory Weissman, Federico Suprani, Kamryn Toney, Houtan Afshar, Noah Stapper, Vanessa Tello, Louise Stolz, Mohsen Poorganji, Zafiris Daskalakis, Lawrence Appelbaum, Jordan Kohn

**Affiliations:** SDSU/UC San Diego; University of California San Diego

## Abstract

Identifying predictors of treatment response to repetitive transcranial magnetic stimulation (rTMS) remain elusive in treatment-resistant depression (TRD). Leveraging electronic medical records (EMR), this retrospective cohort study applied supervised machine learning (ML) to sociodemographic, clinical, and treatment-related data to predict depressive symptom response (>50% reduction on PHQ-9) and remission (PHQ-9 < 5) following rTMS in 232 patients with TRD (mean age: 54.5, 63.4% women) treated at the University of California, San Diego Interventional Psychiatry Program between 2017 and 2023. ML models were internally validated using nested cross-validation and Shapley values were calculated to quantify contributions of each feature to response prediction. The best-fit models proved reasonably accurate at discriminating treatment responders (Area under the curve (AUC): 0.689 [0.638, 0.740], p < 0.01) and remitters (AUC 0.745 [0.692, 0.797], p < 0.01), though only the response model was well-calibrated. Both models were associated with significant net benefits, indicating their potential utility for clinical decision-making. Shapley values revealed that patients with comorbid anxiety, obesity, concurrent psychiatric medication use, and more chronic TRD were less likely to respond or remit following rTMS. Patients with trauma and former tobacco users were more likely to respond. Furthermore, delivery of intermittent theta burst stimulation and more rTMS sessions were associated with superior outcomes. These findings highlight the potential of ML-guided techniques to guide clinical decision-making for rTMS treatment in patients with TRD to optimize therapeutic outcomes.

## INTRODUCTION

Major depressive disorder (MDD) is a prevalent and debilitating condition characterized by persistent feelings of sadness, hopelessness, and loss of interest or pleasure in daily activities (1). An estimated 322 million individuals worldwide meet criteria for MDD, representing a substantial public health challenge impacting quality-of-life, productivity, and overall well-being (2). While various pharmacological, psychotherapeutic, and neuromodulatory treatments are available, 30–40% of individuals with MDD experience treatment-resistant depression (TRD), defined as non-response to two or more trials of first-line interventions (3). Because MDD is a highly heterogeneous disorder, with 277 possible symptom combinations meeting DSM-5 criteria (4), it is possible that different subtypes or symptom clusters of MDD may respond differentially to various treatments (5). Therefore, aligning optimal treatment strategies to symptom presentations may be valuable for addressing TRD.

Repetitive transcranial magnetic stimulation (rTMS) is an established clinical intervention for the treatment of TRD, with response rates of approximately 40–60%, providing a valuable treatment option for patients not responding to first line psychotherapy and pharmacotherapy (6, 7). rTMS involves the application of repetitive magnetic pulses to the cortex, with the dorsolateral prefrontal cortex (DLPFC) a common target for MDD due to its role in mood regulation, cognitive control, executive functioning, and emotion processing (8). The DLPFC is also implicated in the pathophysiology of depression due to its connections with the limbic system, while acute elevations in mood following rTMS provide further evidence of DLPFC dysfunction in MDD (9–12). Ultimately, rTMS is thought to exert its therapeutic effects through modulation of cortical excitability, neurotransmitter systems, and neuroplastic mechanisms (13), but understanding the predictors of treatment response is crucial for tailoring treatment strategies and enhancing clinical outcomes in individuals with TRD.

In recent years, advances in machine learning (ML) have provided new opportunities for leveraging large-scale datasets, such as electronic medical records (EMR), to improve healthcare delivery and inform clinical decision-making (14). For instance, ML prediction models can be used as structured tools to more accurately stratify patients into subgroups or individualized treatment pathways (15). In psychiatry, particularly in TRD and rTMS, ML can reveal latent multifactorial relationships that conventional methods might overlook. This includes the interplay between depressive phenotypes, their causes, comorbidities, and rTMS parameters. Despite the rapid growth of ML in psychiatric research, the generalizability and clinical utility of many ML models published to date remain poor (16). Motivated by the potential of ML to enhance understanding of rTMS treatment for patients with TRD, this study performed a comprehensive retrospective analysis to investigate demographic, clinical, and treatment-related factors associated with treatment response and remission.

This retrospective approach offers several strengths, including relatively large sample sizes, the ability to capture real-world clinical practices, and identify long-term treatment outcomes (17) without the resource demands of a clinical trial. Specifically, this study explores 1) demographic and clinical characteristics of TRD patients receiving rTMS treatment, 2) various protocols and parameters utilized in rTMS administration, and 3) pre-treatment and treatment-related factors predictive of treatment response or remission. This paper aims to address the following questions: whether ML can predict response or remission following rTMS based on pre-treatment patient features and treatment-related factors better than chance; which features distinguish individuals responding favorably to rTMS from those who do not; and whether the ML models may offer clinical benefit. In generating ML models to predict rTMS treatment response and identifying the features that drive both positive and negative outcomes, this study aims to advance understanding of TRD, optimize treatment strategies, and ultimately improve patient outcomes.

## MATERIALS AND METHODS

### Design.

A retrospective study was conducted, including EMR (Epic, Verona, WI, USA) of TRD patients, collected from the clinical database of the UC San Diego Interventional Psychiatry Program (UCSD-IPP). All contributing procedures comply with the ethical standards of the relevant national and international committees on human subjects’ research and the Helsinki Declaration and were approved by the UCSD Human Research Protections Program.

### Patients.

**Figure 1:**
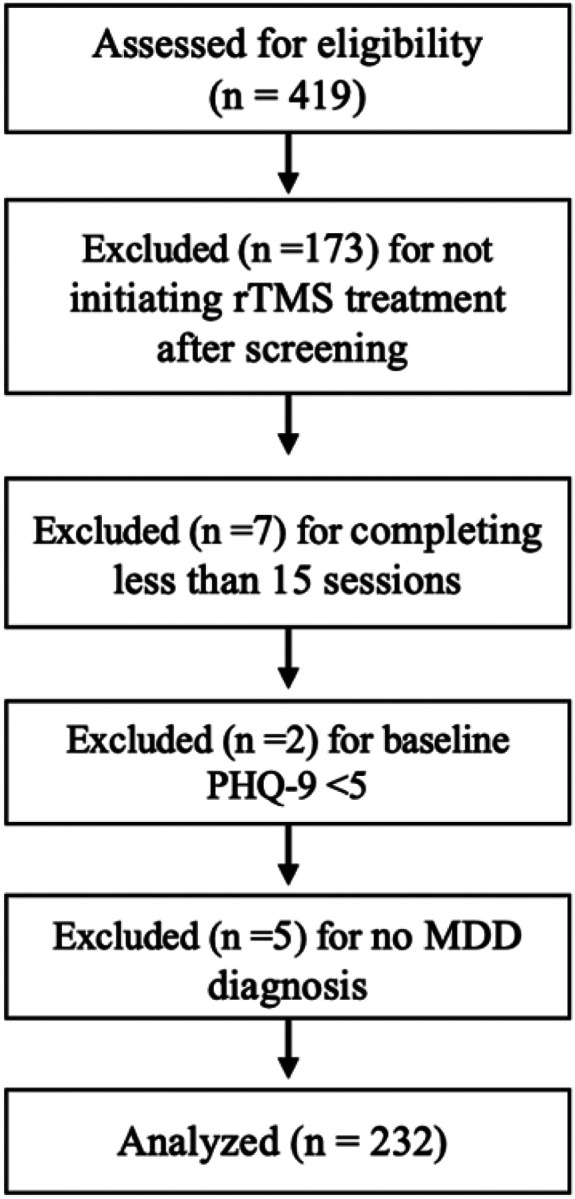
Flowchart of patient inclusion

Included patients were prescribed rTMS treatment from UCSD psychiatrists credentialed in delivering rTMS. rTMS was initiated in outpatients who met criteria for TRD, did not present with any treatment contraindications, had adequate insurance coverage, and provided consent. As illustrated in Fig. 1, EMR records for 419 patients seeking rTMS in 2017–2023 at UCSD were screened for the following inclusion criteria: (i) primary diagnosis of MDD; (ii) aged 18 years and older; (iii) completion of at least 15 sessions by the date of final PHQ-9; (iv) clinically meaningful depressive symptoms, as indicated by PHQ-9 total score > 4; (v) failure to respond to at least 2 antidepressant pharmacotherapy trials; and (vi) completion of clinical evaluations prior to and following the acute treatment phase.

A total of 246 patients began a rTMS treatment course and 239 completed at least 15 sessions before their final PHQ-9 assessment. Two participants had baseline PHQ-9 scores < 5, indicating no clinically significant depressive symptoms, and were excluded from analyses. Five patients were excluded for not having received an MDD diagnosis, yielding a final analytical sample of 232 MDD patients. All patients had previously failed to respond to at least two antidepressant treatments and 90% reported having an MDD diagnosis > 10 years. Eighteen patients had baseline PHQ-9 scores < 10, indicative of minimal depressive symptoms, and response rates within this subgroup were lower than patients with PHQ-9 ≥ 10 at baseline (38.9% vs. 43.9%) but did not significantly differ (X^2^ = 0.17, p = 0.68).

### Treatment procedure.

All rTMS treatment was performed in the UCSD-IPP clinic by technicians trained in protocol administration with oversight by an attending psychiatrist. Overseeing psychiatrists reviewed the treatment plans after each session. rTMS was delivered using either a Magventure MagPro with a B70 coil or a BrainsWay H1-coil system. For all patients, the resting motor threshold (rMT) was identified by finding the motor hotspot and utilizing the lowest level of stimulation intensity to elicit movement in the opposite abductor pollicis brevis (APB) muscle on 50% of stimulation attempts. Once rMT was obtained, treatment session parameters were determined. For Magventure treatments, the coil position was localized using the Beam F3 method, and treatment was delivered per standard practice (18). Patients who were initiated on the iTBS protocol (at 120% rMT), involving 600 total pulses in bursts of 3 with 8-second inter-train-intervals, were transitioned to either BiTBS (90% rMT; 3600 total pulses, 1800 delivered via 3-iTBS and 1800 delivered cTBS 120% rMT protocol to the left and right hemispheres, respectively) or 3-iTBS (iTBS delivered three times consecutively, per Li et al 2014 (19) if there was deemed poor efficacy or issues with tolerability). Standard high-frequency left (HFL; 19 minutes) and low-frequency right (LFR; 10 minutes) protocols were used less commonly, both at 120% rMT. The obsessive-compulsive disorder (OCD) protocol uses a B70 coil and delivers 2000 pulses per session via 50, 2-second trains to dorsomedial prefrontal cortex (DMPFC), at 100% rMT of the foot with the Magventure B70 coil. For the BrainsWay H1-coil system either iTBS or the standard FDA-approved 18 Hz protocol was used at 120% rMT. The end of each patient’s acute treatment period was clinician-determined and coincided with reduced frequency of rTMS treatment to < 2 sessions per week. Sequencing of TMS protocols across all patients included in the analysis is depicted in **Supplemental Fig. 1**.

### Clinical assessment.

The PHQ-9 is a nine-item self-report scale used to assess the presence and severity of depressive symptoms based on the Diagnostic and Statistical Manual (DSM) criteria for a Major Depressive Episode (MDE). Questions are on a four-point scale related to the frequency of symptoms presented on each item, including “not at all,” “several days,” “more than half the days,” and “nearly every day.” Total scores are calculated by adding scores to all nine items (total range: 0–27), and previously established cutoff scores for severity are: 0–4 (no symptoms), 5–9 (mild symptoms), 10–14 (moderate symptoms), 15–19 (moderately severe symptoms), and 20–27 (severe symptoms). The PHQ-9 was administered pre-treatment and at rTMS visits throughout the acute treatment phase to monitor changes in depressive symptoms. Only pre-treatment and post-acute treatment phase scores were used in the present analyses.

### Predictive features.

#### Pre-treatment features.

A total of 32 EMR-derived features were extracted from patient records, including: age, gender, ethnicity, religious affiliation, educational attainment, employment status, relationship status, body mass index (BMI) category (obesity: ≥ 30 kg/m^2^; overweight: 25 ≤ kg/m^2^ < 30; normal 18.5 < kg/m^2^ < 25), alcohol, tobacco, and cannabis use (each operationalized as 3-level factor: current, former, or never), family history of mood disorders, 1st degree relatives with mood or psychiatric disorder diagnoses, prior ECT treatment, prior rTMS treatment, history of psychological trauma, non-suicidal self-injury (NSSI), history of suicidal ideation (SI) or suicide attempts, psychiatric hospitalizations, and duration of current MDE. Psychiatric comorbidities were also recorded, including anxiety disorders, trauma-related disorders, substance use disorders, OCD, somatic symptom and related disorders, personality disorders, autism spectrum disorders, eating disorders, and neurocognitive disorders. Comorbid diagnoses were extracted from physician notes. Psychiatric medication use, including benzodiazepines, non-benzodiazepine sedatives, anxiolytics, antipsychotics, and psychostimulants, was recorded, and antidepressant medication burden was translated into daily defined dose (DDD) equivalents.

#### Treatment-related features.

To determine the role of treatment-related factors in depression outcomes, the following five parameters were recorded: protocol type (deep TMS, iTBS, bilateral TBS, or others that included HFL, LFR, 3-iTBS, or OCD protocols); number of total rTMS sessions; cumulative number of pulses received throughout the treatment course; and, whether patients switched rTMS protocols during treatment.

### Statistical analysis.

R statistical software (v.4.3.1) was used for data analysis. Independent sample *t*-tests and chi-square tests were used to compare demographic and clinical characteristics between rTMS non-responders and responders, defined as patients with ≥ 50% decrease in post-treatment PHQ-9 scores from pre-treatment scores. Pairwise post-hoc tests for factors with more than two levels were false discovery rate (FDR)-corrected. To mitigate low variance within categorical variables, variables were combined and/or operationalized as follows: rTMS protocols: 18 Hz deep TMS (dTMS), BiTBS, or iTBS, made up the majority (86.6%) of rTMS protocols in the sample; therefore, other protocols, including 3-iTBS, 3-iTBS/OCD, OCD, and HFL were combined into a single factor level (“Other”). Race/ethnicity: patients were coded as non-Hispanic, white, or “Other”. Psychiatric comorbidities: patients were coded with 1 if they had at least one comorbid diagnosis. Duration of current MDE: patients were coded as < 6 months, 6–12 months, or > 12 months in duration. Patients were binarized indicating completion of a 4-year degree or not. Relationship status was coded as: currently single/unpartnered, partnered/married, or separated/divorced/widowed. Non- antidepressant psychiatric medication use was coded 1 or 0 for each drug category.

To identify pre-treatment patient characteristics and treatment-associated features that best discriminate response and remission groups, a series of classification models were trained using the *caret* package: (i) regularized logistic regression (elastic net regression; ENet); (ii) support vector machine (SVM) with radial basis function; (iii) random forest (RF); and (iv) stochastic gradient boosting machines (GBM). All features were filtered for near-zero-variance (< 5%) and linear combinations. Nested cross-validation was used for hyperparameter tuning and model performance evaluation using *nestedcv* with 10 inner folds and 20 outer folds. RF parameters optimized in the nested grid search included *mtry* (number of variables to split), *splitrule* (quality of each node split), and *min.node.size* (minimum number of samples in a node), and GBM parameters included *interaction.depth* (highest interaction per tree), *n.minobsinnode* (minimum number of samples in a node to split), *n.trees* (total number of trees), and *shrinkage* (learning rate), whereas the default tuning grids in *caret* were applied for ENet and SVM. To mitigate class imbalance in the dataset, Synthetic Minority Over-sampling TEchnique (SMOTE) was applied to equalize the class ratio (1:1).

Feature selection via a univariate filter was performed on each outer fold prior to hyperparameter tuning, and a Relief-based algorithm (ReliefFbestK in *CORElearn* package; k = 10) was applied in parallel for comparison with the univariate filter. Generally, performance was superior for models trained after univariate filtering (**Supplemental Fig. 3**), with 10 features selected on average (range: 7–13) for all models. Optimization criteria for each model’s hyperparameter tuning procedure were receiver operating characteristic area under the curve (ROC AUC). Continuous features were standardized, and categorical features were one-hot encoded, with the first level dropped as reference. Missing variables were treated as missing completely at random and nonparametric random forest-based imputation was performed using *missForest*.

Generalization performance and 95% confidence intervals were generated by averaging AUC across the best tune of all outer folds. Predicted probabilities across all outer folds were merged to generate additional performance metrics (F-score, sensitivity, specificity) and models with the best performance for an outcome were selected for downstream analysis. Significance of AUC values were determined by permutation testing (B = 99) of class labels, re-running the procedure for each model, and calculating the proportion of permuted models with AUC values greater than the observed data. Alpha level was set at 0.05. The contributions of predictive features were determined using SHapley Additive exPlanations (SHAP) analysis in the *fastshap* R package, and directionality of associations between features and outcome variables was ascertained by visualizing individual SHAP values and their dependencies with the *shapviz* package in R.

Using the best performing models, the predicted probabilities of treatment response and remission were derived. Model calibration was assessed with the Hosmer-Lemeshow (HL) goodness-of-fit test by binning predicted probabilities into deciles. Probability estimates were updated using beta regression, which has been shown to outperform other calibration methods (20), and recalibrated probabilities were reassessed using HL test. Decision curve analysis (DCA) was applied using *dcurves* to assess the clinical utility of prediction models through estimation of net benefit across thresholds of predicted risks using recalibrated probabilities from both “response” and “remission” classification models. Net benefit is equivalent to the percentage of individuals appropriately treated (“true positives”, responders and remitters) minus a weighted percentage of those inappropriately treated (“false positives”).

## RESULTS

### Patient and treatment-related characteristics

**Figure 2:**
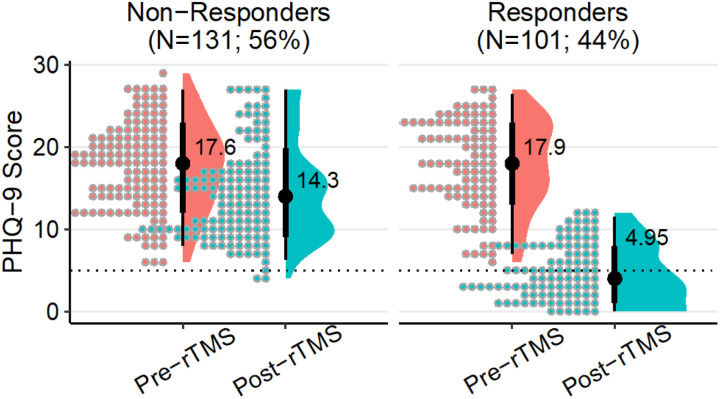
Depression scores pre- and post-rTMS treatment course. Each point represents an individual patient’s PHQ-9 score at baseline and the conclusion of their acute treatment phase, grouped by response status. Horizontal dashed line at PHQ-9 = 5 indicates the threshold for depression remission.

Sociodemographic and clinical characteristics of patients, and treatment-related characteristics included in the analysis are outlined in Table 1. Across all patients, post-treatment PHQ-9 scores were significantly reduced compared to pre-treatment values (mean difference = −7.56, *t*_231_ = −17.6, *d* = 1.16, 95% CI = 0.99–1.32). In total, 101 patients (44%) responded to rTMS, (mean difference = −13.0, t_100_ = −29.4, *d* = 2.94, 95% CI = 2.48–3.39; Fig. 2) and 53 patients (23%) remitted. The average patient age was 55.1±16.9 and 54.1±17.0 years in the responder and non-responder groups, respectively. Patient sex and ethnicity did not differ between groups; however, responders were more likely to have been former smokers than non-responders, and less likely to have never used alcohol. Most patients were naïve to rTMS (88.4%) and ECT (83.3%) treatment, and the majority (60%) had MDEs lasting longer than 12 months at treatment outset. Baseline PHQ-9 scores did not differ between responders (17.9±5.4) and non-responders (17.6±5.3). Responders were more likely to have a history of trauma and to be diagnosed with trauma disorder compared to non-responders, but were less likely to have an anxiety disorder or be prescribed benzodiazepines or antipsychotic medications. The ratio of patients receiving sequential iTBS-BiTBS to iTBS-only treatment was higher among non-responders than responders (p_adj_ = 0.04), and non-responders received fewer rTMS sessions on average compared to responders.

### Classification results

#### Treatment Response.

**Figure 3:**
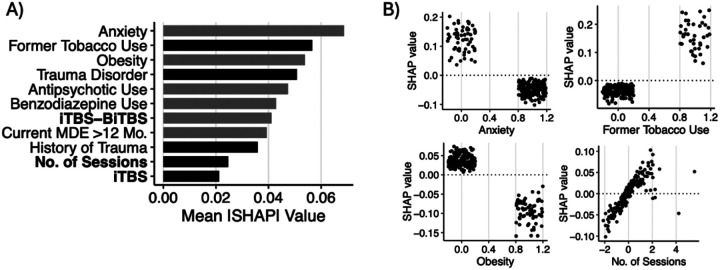
A) Ranked feature importance for treatment response classification model. Grey bars indicate negative association with treatment response (worse outcome), and black bars indicate positive association (better outcome). B) Partial dependence plots illustrating directional associations between selected features and treatment response. Larger positive SHAP values indicate greater probabilities of treatment response, whereas more negative values indicate lower probabilities of response.

Across the four models tested, the SVM model predicted treatment response significantly above chance (p < 0.01; **Supplemental Fig. 2**) with modest discrimination performance (mean AUC = 0.689, 95% CI = 0.638–0.740), outperforming RF (0.654, 0.594–0.715), ENet (0.668, 0.616–0.719), and GBM (0.617, 0.563–0.671) models (**Supplemental Fig. 3**). Thus, the SVM model was evaluated further. The sensitivity and specificity of the model to discriminate treatment responders from non-responders were 72.3% and 53.4%, respectively, with an F-score of 0.64. Following recalibration, observed and predicted probabilities of treatment response were similar (HL test: $\chi_{8}^{2}=5.51$, p=0.70, RMSE=1.73; **Supplemental Fig. 4**). In descending order, the most important variables in prediction of treatment response were anxiety disorder, former tobacco use, obesity, trauma disorder, antipsychotic use, benzodiazepine use, iTBS-BiTBS protocol, > 12-month duration of current MDE, history of trauma, number of rTMS sessions, and iTBS protocol (Fig. 3A). Partial dependence plots illustrating the relationship between the top-3 features, the total number of rTMS sessions, and treatment response are shown in Fig. 3B. DCA indicated that for threshold probabilities between 25% and 55%, corresponding to a number needed-to-treat (NNT) between 4 and 2 respectively, the net benefit of applying the model to aid in determining whether a patient should undergo rTMS is higher than either a “treat all” or “treat none” approach (Fig. 5A).

#### Depression remission.

**Figure 4:**
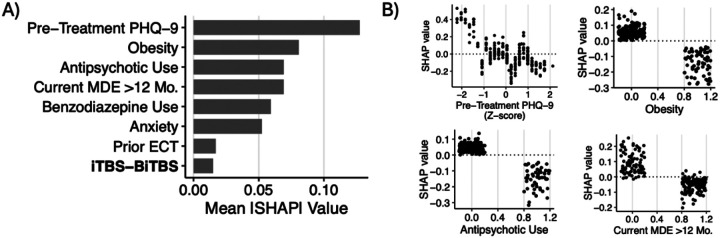
A) Ranked feature importance for depression remission classification model. Grey bars indicate negative association with remission (worse outcome). B) Partial dependence plots illustrating directional associations between selected features and depression remission, indicated by SHAP values.

**Figure 5:**
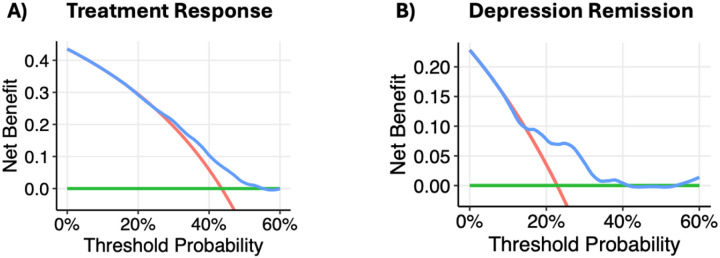
Decision curve analysis for recalibrated A) treatment response and B) depression remission models. Threshold probability represents the point at which a positive response is equally valued to avoiding unnecessary treatment. Decision curves for each model are shown in blue, compared to a “treat all” approach shown in red and a “treat none” approach shown in green.

Across the four models tested, the GBM model predicted remission following rTMS treatment significantly above chance (p < 0.01; **Supplemental Fig. 2**) with good discrimination performance (mean AUC = 0.745, 95% CI = 0.692–0.797), outperforming RF (0.735, 0.680–0.791), ENet (0.740, 0.668–0.813), and SVM (0.683, 0.627–0.738) models (**Supplemental Fig. 3**). Thus, the GBM model was evaluated further. The sensitivity and specificity of the model to discriminate remitters from non-remitters were 58.5% and 77.7%, respectively, with an F-score of 0.50. Following recalibration, the observed and predicted probabilities of treatment response remained dissimilar (HL test: $\chi_{8}^{2}=19.1$, p=0.01, RMSE=2.73; **Supplemental Fig. 4**). In descending order, the most important variables in the prediction of depression remission were pre-treatment PHQ-9 score, obesity, antipsychotic use, current MDE greater than 12 months, benzodiazepine use, anxiety, prior ECT, and iTBS-BiTBS protocol (Fig. 4A). Partial dependence plots illustrating the relationship between the top-four features and treatment response are shown in Fig. 4B. DCA indicated that for threshold probabilities between 15% and 40%, corresponding to a NNT between approximately 7 and 3, respectively, using the model to determine whether a patient should undergo rTMS yielded greater net benefit than employing either a “treat all” or “treat none” strategy (Fig. 5).

## DISCUSSION

Findings presented in this study underscore the substantial variability in depression responses and remission, despite rTMS efficacy, and the promise of ML to predict treatment outcomes using EMR data alone. Importantly, both ML models for response and remission identified psychiatric comorbidities, depressive episode duration, and specific rTMS protocols as predictive features, with trauma history also associated with response outcomes.

### Predictive features of treatment outcomes:

Past research investigating the relationship between psychiatric comorbidities and treatment response has yielded valuable insights into the complex interplay between mental health conditions and their respective treatments (21). This literature presents mixed results regarding impact of trauma history on rTMS. Experiences of childhood trauma and PTSD diagnosis have been reported to negatively affect rTMS outcomes in MDD patients (22, 23) or to have no significant impact (24). Positive findings, as presented here, may point to a consequence of trauma-induced neuroplastic changes that sensitize the brain to neurostimulation (25). For example, the therapeutic impact of rTMS could be modulated by individual differences in neural circuitry affected by trauma, which are not always uniformly engaged by standard rTMS protocols, such as 10 Hz rTMS to the left DLPFC as done in Hu et al. (2012) and Yesavage et al. (2018).

Former tobacco use was identified as a positive predictor of treatment response. Past literature on tobacco use in rTMS outcomes is mixed (26, 27), but nicotine’s complex neurobiological effects may enhance efficacy. Nicotine potentiates dopaminergic neurotransmission via nicotinic acetylcholine receptor binding on dopaminergic neurons in the mesolimbic pathway associated with mood regulation (28). This dopamine surge may have a persistent effect that synergizes with rTMS mechanisms, inducing changes in brain plasticity (29). Coupled with trauma history, these associations suggest past experiences and lifestyle modifications may prime the brain’s receptivity to rTMS.

Features including comorbid anxiety disorder, concomitant benzodiazepine or antipsychotic medications, and a longer duration of current MDE were linked to lower probabilities of treatment response and remission. Anxiety is highly comorbid with depression, and this specific comorbidity may reflect distinct neurobiological underpinnings, affecting the response to rTMS. For instance, hyperactivity in the amygdala and its connectivity with the PFC, can be more pronounced in individuals with comorbid anxiety (30). An extensive body of research supports that comorbid anxiety can complicate the course of depression, often leading to a more chronic and treatment-resistant condition (31).

Concurrent use of benzodiazepines or antipsychotics was associated with less favorable responses. These medications exert sedative effects and reduce cortical excitability, potentially impeding operating mechanisms of rTMS. Benzodiazepines, for instance, enhance GABAergic inhibition, which may diminish excitatory processes (32). Antipsychotics, particularly those blocking dopamine receptors, have been shown to attenuate rTMS outcomes (33), potentially disrupting the dopaminergic modulation crucial for rTMS-induced neuroplasticity. These findings suggest patients prescribed benzodiazepines or antipsychotics may require adjusted protocols, perhaps with increased intensity or frequency to overcome medication-induced reductions in cortical excitability, or timing treatment sessions relative to medication dosing.

The observation that longer depressive episodes correlated with poorer outcomes is consistent research indicating depression chronicity can negatively impact the efficacy of various treatments. Chronic depression is associated with more severe neurobiological changes, including alterations in the hippocampus and PFC (34). Earlier intervention might enhance response by addressing such changes before they become entrenched. Fava (2003) highlighted the predictive role of untreated depression duration in treatment resistance (35) and underscoring the possibility that adjusting standard protocols, or combining with other interventions, may increase efficacy for these patients.

The observed absence of sociodemographic variables, such as age, sex, and ethnicity, impacting outcomes aligns with past studies (36), yet diverges from others. For example, Hanlon and colleagues (2022) found younger age and female sex were associated with better rTMS response rates, suggesting demographic characteristics may influence treatment success (37). The contrasting evidence indicates a complex relationship between sociodemographic and rTMS efficacy, warranting further investigation to clarify their roles in treatment personalization and optimization.

Accrued effects from patient history, such as trauma and anxiety, and concurrent benzodiazepine use may influence rTMS outcomes. The potential cumulative effect of these factors on response could be linked to their disparate impact on brain neuroplasticity and neurotransmission. For instance, a history of trauma and relatively lower anxiety might enhance receptivity to rTMS via induced neuroplastic changes, whereas the suppressive effect of benzodiazepines on cortical excitability could counteract these benefits, requiring adjustments in treatment protocols to optimize efficacy. Further investigation into the interaction of these factors may provide deeper insights into personalized rTMS strategies for patients with complex clinical profiles.

Regarding treatment-related factors, the number of rTMS sessions was a positive predictor, with SHAP values indicating a positive linear dose-response relationship. This relationship aligns with notions that additional sessions sustain antidepressant effects, possibly through cumulative neuroplastic changes. However, this is not consistently upheld, as a meta-analysis reported higher doses of rTMS, measured by total pulses, were not always associated with depression improvement (38). Adherence to the iTBS protocol was also among the strongest predictors of response. Notably, iTBS demonstrated superiority over iTBS-BiTBS in our sample, challenging some of the existing literature that suggests bilateral approaches can be advantageous for certain patient populations (39). Despite this, the current findings likely relate to iTBS-BiTBS patients experiencing greater treatment-resistance at baseline, with providers transitioning protocols from unilateral to bilateral in cases of inadequate symptom relief.

### Machine learning, electronic medical records, and clinical decision-making

Recent reviews of ML prediction models in psychiatric research have found that despite relatively ‘good’ discrimination performance (AUC ~ 0.65–0.80), most studies had methodological weaknesses that limited their generalizability and utility in clinical decision-making (16, 40). The authors recommended more comprehensive reporting of ML methods and results, more robust validation methods, model calibration, and formal assessment of clinical utility, such as DCA (40, 41). In our analyses, DCA demonstrated that the response and remission models outperformed “treat all” and “treat none” strategies within probability threshold ranges of 25–55% and 15–40%, or NNT between 2 and 4 patients, and 3 and 7 patients, respectively. Both models indicate net clinical benefit within their respective threshold ranges, though more refined models incorporating additional patient features, such as neurobiological markers, polygenic risk scores, and psychosocial determinants, may confer additional benefit.

The implications of these findings are multi-fold. Firstly, they re-affirm the clinical utility of rTMS in managing TRD, especially when considering individual patient histories and comorbidities. Secondly, these results lend support for the predictive power of ML algorithms derived from EMR for refining treatment approaches, suggesting that these tools could become integral to psychiatric precision medicine. Future research should aim to validate these predictive models in diverse clinical settings, ensuring that findings are replicable and robust.

### Limitations.

The retrospective study design provides a robust dataset but holds inherent limitations. Firstly, these ML models were not externally validated in an independent sample. However, nested cross-validation, a robust internal validation procedure, provides a strong estimation of model generalizability. Secondly, this study is susceptible to selection bias since it involves patients who initiated and completed treatment, which may exclude those with different response trajectories, possibly skewing findings towards patients more likely to complete treatment. Moreover, the sample in this study was predominantly highly educated and non-Hispanic White, limiting generalization to other demographics and pointing more towards the need to address disparities in access to TMS (42). Third, information bias may arise from EMR data availability, accuracy, and completeness, with additional variability in the timing of data collection vis-à-vis treatment, possibly creating discrepancies between recorded and actual patient conditions. Future research should address these limitations by implementing prospective designs, standardizing rTMS protocols, timely data collection, and diverse populations.

## CONCLUSION

This study elucidates predictors of treatment response to rTMS in TRD patients and highlights the importance of treatment approaches tailored to individual characteristics. By leveraging EMR and ML techniques, this study identified demographic, clinical, and treatment-related factors associated with outcomes, providing valuable insights for clinical practice and research.

## Figures and Tables

**Table 1:** Sociodemographic and clinical characteristics of patient sample.

	All patients (n = 232)	Responders (n = 101; 43.5%)	Non-Responders (n = 131; 56.5%)	Test Statistic
**Sociodemographic characteristics**				
Age (yrs)	54.5 (16.9)	55.1 (16.9)	54.1 (17.0)	t_198_ 113, P = 0.26
Sex (%F)	63.4%	62.4%	64.1%	$\chi_{1}^{2}=0.02$, p = 0.89
Ethnicity (%NHW/B/H/A/P/MO)	76/1/6/4/1/12	75/1/4/5/2/13	76/2/7/3/0/12	$\chi_{6}^{2}=5.43$, p = 0.49
Religiously affiliated (%)	23.9%	18.2%	28.3%	$\chi_{1}^{2}=316$, p = 0.08
Relationship status (S/P/SW)	35 / 48 / 17	39 / 46 / 15	31 / 50 / 19	$\chi_{1}^{2}=159$, p = 0.45
College graduate (%)	81.6%	79.8%	82.9%	$\chi_{1}^{2}=037$, p = 0.54
BMI category (%Nm / Ow / Ob)	40 / 30 / 30	48 / 29 / 23	34 / 31 / 35	$\chi_{2}^{2}=5.79$, p = 0.06
1st -degree relative w/psych dx (%)	77.4%	81.9%	74.0%	$\chi_{1}^{2}=192$, p = 0.17
**Tobacco use (% N/C/F)**	**75 / 7 / 18**	**63 / 9 / 28**	**85 / 5 / 10**	**$\backslash \text{varvec}\chi_{2}^{2}=14.7$, p < 0.01**
Cannabis use (% N/C/F)	67 / 22 / 11	63 / 25 / 12	69 / 20 / 11	$\chi_{1}^{2}=101$, p = 0.60
**Alcohol use (% N/C/F)**	**37 / 46 / 17**	**26 / 52 / 22**	**45 / 41 / 14**	**$\backslash \text{varvec}\chi_{1}^{2}=10.1$, p < 0.01**
**Clinical characteristics**				
Psychiatric comorbidities (n)	0.23 (0.4)	0.23 (0.4)	0.24 (0.4)	*t*_216_=0.16, p = 0.87
Antidepressant drug use (DDD)	1.75 (1.7)	1.65 (1.5)	1.83 (1.8)	*t*_229_=0.79, p = 0.43
Prior rTMS treatment	11.6%	15.8%	8.4%	$\chi_{1}^{2}=3.07$, p = 0.08
Baseline PHQ-9 score	17.8 (5.3)	17.9 (5.4)	17.6 (5.3)	*t*_213_=0.42, p = 0.67
MDE duration (< 6/6–12/12 + mo)	21%/18%/60%	28%/19%/53%	16%/18%/66%	$\chi_{2}^{2}=4.76$, p = 0.09
Prior ECT	17.7%	13.9%	20.6%	$\chi_{1}^{2}=1.79$, p = 0.18
**History of trauma**	**45.3%**	**54.5%**	**38.2%**	**$\backslash \text{varvec}\chi_{1}^{2}=6.11$, p = 0.01**
Prior psych hospitalization	35.8%	41.6%	31.3%	$\chi_{1}^{2}=2.63$, p = 0.11
Suicidal ideation history	65.1%	66.3%	64.1%	$\chi_{1}^{2}=0.12$, p = 0.73
Non-suicidal self-injury history	11.2%	9.9%	12.2%	$\chi_{1}^{2}=0.31$, p = 0.58
**Anxiety disorder**	**72.8%**	**65.3%**	**78.6%**	**$\backslash \text{varvec}\chi_{1}^{2}=5.08$, p = 0.02**
Prior suicide attempt	25.9%	29.7%	22.9%	$\chi_{1}^{2}=1.38$, p = 0.24
Substance use disorder	14.2%	14.9%	13.7%	$\chi_{1}^{2}=0.06$, p = 0.81
ADHD diagnosis	15.5%	19.8%	12.2%	$\chi_{1}^{2}=2.51$, p = 0.11
**Trauma disorder diagnosis**	**22.4%**	**31.7%**	**15.3%**	$\backslash \text{varvec}\chi_{1}^{2}=8.84$, **p < 0.01**
**Benzodiazepine use**	**29.0%**	**21.8%**	**34.4%**	$\backslash \text{varvec}\chi_{1}^{2}=4.20$, **p = 0.04**
**Antipsychotic medication use**	**24.2%**	**17.0%**	**29.8%**	$\backslash \text{varvec}\chi_{1}^{2}=5.04$, **p = 0.02**
Psychostimulant medication use	19.5%	20.0%	19.1%	$\chi_{1}^{2}=0.03$, p = 0.86
Non-benzodiazepine sedative use	25.5%	27.0%	24.4%	$\chi_{1}^{2}=0.20$, p = 0.66
Mood stabilizer use	18.2%	14.0%	21.4%	$\chi_{1}^{2}=2.07$, p = 0.15
**Treatment-related characteristics**				
**TMS protocol (% BiTBS/dTMS/iTBS/iTBS-BiTBS/Other)**	**16/36/10/25/13**	**13/41/16/18/13**	**18/33/5/31/14**	$\backslash \text{varvec}\chi_{4}^{2}=11.8$, **p = 0.02**
**Total number of rTMS sessions**	**37.4 (10.6)**	**39.1 (10.8)**	**36.2 (10.2)**	$\backslash \text{varvec}t_{209}=2.08$, **p = 0.04**
Cumulative rTMS pulses	88,804(44,862)	88,832(48,831)	88,782(41,738)	*t*_196_=0.01, p = 0.99
rTMS protocol switch (%)	58.2%	53.5%	61.8%	$\chi_{1}^{2}=1.64$ = 1.64, p = 0.20
Weeks from final patient in DB	136 (81)	146 (85)	128 (78)	*t*_204_=162, p = 0.11
Resting motor threshold (%)	46.0%	46.3%	45.7%	*t*_200_=0.44, p = 0.66
Length of treatment (days)	66.3 (19.9)	69.0 (19.5)	64.2 (19.9)	*t*_217_=1.85, p = 0.07

Other rTMS protocols include 3-iTBS, 3-iTBS/OCD, HFL, and OCD. Ethnicities reported include Non-Hispanic White (NHW), Black (B), Hispanic (H), Asian (A), Persian (P), and Mixed or Other (MO).Substance use reported as never (N), current (C), or former (F). Relationship status reported as Single (S), Partnered/Married (P), or Widowed/Divorced (W). Psychiatric comorbidities included OCD, somatic symptom disorders, personality disorders, autism spectrum disorders, eating disorders, and neurocognitive disorders. BMI categories corresponded to kg/m^2^ ≥ 30 (Ob), 25 ≤ Ow < 30, and 18.5< Nm < 25.

## References

[1] American Psychiatric Association, American Psychiatric Association, editors. Diagnostic and statistical manual of mental disorders: DSM-5. 5th ed. Washington, D.C: American Psychiatric Association; 2013. 947 p.

[2] (WHO) WHO. Mental disorders [Internet]. [cited 2023 Jun 5]. Available from: https://www.who.int/news-room/fact-sheets/detail/mental-disorders

[3] Zhdanava M, Pilon D, Ghelerter I, Chow W, Joshi K, Lefebvre P, The Prevalence and National Burden of Treatment-Resistant Depression and Major Depressive Disorder in the United States. J Clin Psychiatry. 2021;82(2):0–0. 10.4088/JCP.20m13699 33989464

[4] Zimmerman M, Ellison W, Young D, Chelminski I, Dalrymple K. How many different ways do patients meet the diagnostic criteria for major depressive disorder? Compr Psychiatry. 2015;56:29–34. 25266848 10.1016/j.comppsych.2014.09.007

[5] Lynch CJ, Gunning FM, Liston C. Causes and Consequences of Diagnostic Heterogeneity in Depression: Paths to Discovering Novel Biological Depression Subtypes. Biol Psychiatry. 2020;88(1):83–94. 32171465 10.1016/j.biopsych.2020.01.012

[6] Benster LL, Weissman CR, Stolz LA, Daskalakis ZJ, Appelbaum LG. Pre-clinical indications of brain stimulation treatments for non-affective psychiatric disorders, a status update. Transl Psychiatry. 2023;13(1):1–12. 38097566 10.1038/s41398-023-02673-2 PMC10721798

[7] Aaronson S. Overview of a large transcranial magnetic stimulation patient outcomes database. Brain Stimul Basic Transl Clin Res Neuromodulation. 2021;14(6):1717.

[8] Fitzgerald PB, Oxley TJ, Laird AR, Kulkarni J, Egan GF, Daskalakis ZJ. An analysis of functional neuroimaging studies of dorsolateral prefrontal cortical activity in depression. Psychiatry Res Neuroimaging. 2006;148(1):33–45. 10.1016/j.pscychresns.2006.04.006 17029760

[9] Mayberg HS. Limbic-cortical dysregulation: a proposed model of depression. J Neuropsychiatry Clin Neurosci. 1997;9(3):471–81. 9276848 10.1176/jnp.9.3.471

[10] George MS, Lisanby SH, Avery D, McDonald WM, Durkalski V, Pavlicova M, Daily left prefrontal transcranial magnetic stimulation therapy for major depressive disorder: a sham-controlled randomized trial. Arch Gen Psychiatry. 2010;67(5):507–16. 20439832 10.1001/archgenpsychiatry.2010.46

[11] Cole EJ, Stimpson KH, Bentzley BS, Gulser M, Cherian K, Tischler C, Stanford Accelerated Intelligent Neuromodulation Therapy for Treatment-Resistant Depression. Am J Psychiatry. 2020;177(8):716–26. 32252538 10.1176/appi.ajp.2019.19070720

[12] Chung SW, Hoy KE, Fitzgerald PB. Theta-burst stimulation: a new form of TMS treatment for depression? Depress Anxiety. 2015;32(3):182–92. 25450537 10.1002/da.22335

[13] Peng Z, ZHOU C, XUE S, BAI J, YU S, LI X, Mechanism of Repetitive Transcranial Magnetic Stimulation for Depression. Shanghai Arch Psychiatry. 2018;30(2):84–92. 29736128 10.11919/j.issn.1002-0829.217047 PMC5936045

[14] Beam AL, Kohane IS. Big Data and Machine Learning in Health Care. JAMA. 2018;319(13):1317–8. 29532063 10.1001/jama.2017.18391

[15] Rajkomar A, Dean J, Kohane I. Machine Learning in Medicine. N Engl J Med. 2019;380(14):1347–58. 30943338 10.1056/NEJMra1814259

[16] Meehan AJ, Lewis SJ, Fazel S, Fusar-Poli P, Steyerberg EW, Stahl D, Clinical prediction models in psychiatry: a systematic review of two decades of progress and challenges. Mol Psychiatry. 2022;27(6):2700–8. 35365801 10.1038/s41380-022-01528-4 PMC9156409

[17] Rassen JA, Bartels DB, Schneeweiss S, Patrick AR, Murk W. Measuring prevalence and incidence of chronic conditions in claims and electronic health record databases. Clin Epidemiol. 2018;11:1–15. 30588119 10.2147/CLEP.S181242 PMC6301730

[18] Beam W, Borckardt JJ, Reeves ST, George MS. An efficient and accurate new method for locating the F3 position for prefrontal TMS applications. Brain Stimulat. 2009;2(1):50–4. 10.1016/j.brs.2008.09.006 PMC2882797 20539835

[19] Li CT, Chen MH, Juan CH, Huang HH, Chen LF, Hsieh JC, Efficacy of prefrontal theta-burst stimulation in refractory depression: a randomized sham-controlled study. Brain J Neurol. 2014;137(Pt 7):2088–98. 10.1093/brain/awu109 24817188

[20] Ojeda FM, Jansen ML, Thiéry A, Blankenberg S, Weimar C, Schmid M, Calibrating machine learning approaches for probability estimation: A comprehensive comparison. Stat Med. 2023;42(29):5451–78. 37849356 10.1002/sim.9921

[21] Krishnan KRR. Comorbidity and depression treatment. Biol Psychiatry. 2003;53(8):701–6. 12706956 10.1016/s0006-3223(02)01787-0

[22] Yesavage JA, Fairchild JK, Mi Z, Biswas K, Davis-Karim A, Phibbs CS, Effect of Repetitive Transcranial Magnetic Stimulation on Treatment-Resistant Major Depression in US Veterans. JAMA Psychiatry. 2018;75(9):884–93. 29955803 10.1001/jamapsychiatry.2018.1483 PMC6142912

[23] Hu YT, Hu XW, Han JF, Zhang JF, Wang YY, Wolff A, Childhood trauma mediates repetitive transcranial magnetic stimulation efficacy in major depressive disorder. Eur Arch Psychiatry Clin Neurosci. 2021;271(7):1255–63. 34117915 10.1007/s00406-021-01279-3

[24] Ng E, Wong EHY, Lipsman N, Nestor SM, Giacobbe P. Adverse childhood experiences and repetitive transcranial magnetic stimulation outcomes for depression. J Affect Disord. 2023;320:716–24. 36206889 10.1016/j.jad.2022.09.153

[25] Karsen EF, Watts BV, Holtzheimer PE. Review of the Effectiveness of Transcranial Magnetic Stimulation for Post-traumatic Stress Disorder. Brain Stimul Basic Transl Clin Res Neuromodulation. 2014;7(2):151–7. 10.1016/j.brs.2013.10.006 24486424

[26] Abdelrahman AA, Noaman M, Fawzy M, Moheb A, Karim AA, Khedr EM. A double-blind randomized clinical trial of high frequency rTMS over the DLPFC on nicotine dependence, anxiety and depression. Sci Rep. 2021;11(1):1640. 33452340 10.1038/s41598-020-80927-5 PMC7810712

[27] Poulet E, Galvao F, Haffen E, Szekely D, Brault C, Haesebaert F, Effects of smoking status and MADRS retardation factor on response to low frequency repetitive transcranial magnetic stimulation for depression. Eur Psychiatry. 2016;38:40–4. 27657664 10.1016/j.eurpsy.2016.04.004

[28] Benowitz NL. Pharmacology of Nicotine: Addiction, Smoking-Induced Disease, and Therapeutics. Annu Rev Pharmacol Toxicol. 2009;49:57–71. 18834313 10.1146/annurev.pharmtox.48.113006.094742 PMC2946180

[29] Belujon P, Grace AA. Dopamine System Dysregulation in Major Depressive Disorders. Int J Neuropsychopharmacol. 2017;20(12):1036–46. 29106542 10.1093/ijnp/pyx056 PMC5716179

[30] Shin LM, Liberzon I. The neurocircuitry of fear, stress, and anxiety disorders. Neuropsychopharmacol Off Publ Am Coll Neuropsychopharmacol. 2010;35(1):169–91. 10.1038/npp.2009.83 PMC3055419 19625997

[31] Comorbid anxiety and depression in adults: Epidemiology, clinical manifestations, and diagnosis -UpToDate [Internet]. [cited 2024 Mar 3]. Available from: https://www.uptodate.com/contents/comorbid-anxiety-and-depression-in-adults-epidemiology-clinical-manifestations-and-diagnosis

[32] Wilson S, Olsen S, Sullivan C, Cooper D, Somez I, Widge AS, Concurrent Benzodiazepine Use and TMS Clinical Outcomes. Biol Psychiatry. 2021;89(9):S288.

[33] Hebel T, Abdelnaim M, Deppe M, Langguth B, Schecklmann M. Attenuation of antidepressive effects of transcranial magnetic stimulation in patients whose medication includes drugs for psychosis. J Psychopharmacol Oxf Engl. 2020;34(10):1119–24. 10.1177/0269881120922965 PMC7543016 32522071

[34] Sheline YI, Gado MH, Kraemer HC. Untreated depression and hippocampal volume loss. Am J Psychiatry. 2003;160(8):1516–8. 12900317 10.1176/appi.ajp.160.8.1516

[35] Fava M. Diagnosis and de nition of treatment-resistant depression. Biol Psychiatry. 2003;53(8):649–59. 12706951 10.1016/s0006-3223(03)00231-2

[36] Leuchter M, Citrenbaum C, Wilson A, Tibbe T, Jackson N, Krantz D, Holding Steady: Age-Related Effects on Treatment Response During rTMS Treatment of Depression. Am J Geriatr Psychiatry. 2023;31(3, Supplement):S119–20.

[37] Hanlon CA, McCalley DM. Sex/Gender as a Factor That Influences Transcranial Magnetic Stimulation Treatment Outcome: Three Potential Biological Explanations. Front Psychiatry. 2022;13:869070. 35573331 10.3389/fpsyt.2022.869070 PMC9098922

[38] Yu CL, Kao YC, Thompson T, Brunoni AR, Hsu CW, Carvalho AF, The association of total pulses with the efficacy of repetitive transcranial magnetic stimulation for treatment-resistant major depression: A dose-response meta-analysis. Asian J Psychiatry. 2024;92:103891. 10.1016/j.ajp.2023.103891 38183740

[39] Blumberger DM, Vila-Rodriguez F, Thorpe KE, Feffer K, Noda Y, Giacobbe P, Effectiveness of theta burst versus high-frequency repetitive transcranial magnetic stimulation in patients with depression (THREE-D): a randomised non-inferiority trial. The Lancet. 2018;391(10131):1683–92. 10.1016/S0140-6736(18)30295-2 29726344

[40] Grzenda A, Widge AS. Electronic health records and stratified psychiatry: bridge to precision treatment? Neuropsychopharmacol Off Publ Am Coll Neuropsychopharmacol. 2024;49(1):285–90. 10.1038/s41386-023-01724-y PMC10700348 37667021

[41] Vickers AJ, Calster BV, Steyerberg EW. Net benefit approaches to the evaluation of prediction models, molecular markers, and diagnostic tests. BMJ. 2016;352:i6. 26810254 10.1136/bmj.i6 PMC4724785

[42] Nagra DS, Stolz LA, Weissman CR, Appelbaum LG. Access to interventional psychiatric treatments in the United States: Disparities and proposed solutions. Glob Health Econ Sustain. 2024;2(1):2456.

